# Agriculture is adapting to phenological shifts caused by climate change, but grassland songbirds are not

**DOI:** 10.1002/ece3.7548

**Published:** 2021-05-18

**Authors:** Maeve M. McGowan, Noah G. Perlut, Allan M. Strong

**Affiliations:** ^1^ School of Marine and Environmental Programs University of New England Biddeford ME USA; ^2^ Rubenstein School of Environment and Natural Resources University of Vermont Burlington VT USA

**Keywords:** bobolink, *Dolichonyx oryzivorus*, El Niño southern oscillation, North Atlantic oscillation, *Passerculus sandwichensis*, savannah sparrow

## Abstract

Migratory birds time their migration based on cues that signal resource availability for reproduction. However, with climate change, the timing of seasonal events may shift, potentially inhibiting the ability of some species to use them as accurate cues for migration. We studied the relationship between phenological shifts and reproduction by long‐ and short‐distance migratory songbirds—Bobolinks (*Dolichonyx oryzivorus)* and Savannah Sparrows (*Passerculus sandwichensis)*. Our study population breeds in hayfields and pastures in Vermont, USA, where farmers are also changing management activities in response to climate change. From 2002 to 2019, we monitored nest initiation dates to quantify correlations with environmental factors and the timing of nest initiation. We collected historical and projected precipitation and temperature data for the breeding grounds, and their respective wintering and stopover sites, the North Atlantic Oscillation (NAO) and the El Niño Southern Oscillation (ENSO). We predicted that winter conditions experienced by the short‐distance migrant, the Savannah Sparrow, but not the long‐distance migrant, the Bobolink, would explain the timing and success of nesting, however that this timing would be misaligned with changes in agricultural practices by hay farmers. Nest initiation dates did not show significant directional change for either species, but did vary among years. Interannual variation in Savannah Sparrow nest initiation dates was best explained by the interaction between precipitation on the breeding grounds and average wintering site (Wilmington, North Carolina). For Bobolinks, interannual variation in nest initiation dates was best explained by the interaction between breeding ground precipitation and average temperature in their fall stopover site (Barquisimieto, Venezuela). However, first haying dates in Vermont advanced by ~10 days over 18 years. These results suggest that the conflict between the timing of hay harvests and grassland songbird reproduction will increase, further threatening population processes for these species, as early harvests notably decrease annual productivity.

## Graphical abstract

Nest initiation date (Julian date) of Savannah Sparrows and Bobolinks (A) and first hay harvest dates (B) from 2002‐2019 in the Champlain Valley of Vermont.

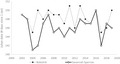

## 1 INTRODUCTION

Migratory birds determine the timing of their migration based on endogenous rhythms as well as various environmental cues, including but not limited to the blooming and fruiting of plants, and the emergence of insects from diapause, as these cues directly signal resource availability for reproduction (Taylor at al., 2016). These cues may be observed on wintering grounds or during spring migration. However, as the climate changes, the timing of seasonal events will likely shift and may do so dramatically (Parmesan, 2007), thus potentially inhibiting the ability of species to use them as accurate cues for migration. However, the degree to which these shifts affect a species’ migration may be based on how reliant that species is on phenological cues. For example, short‐distance migratory birds (those that winter north of the Tropic of Cancer; 23°27′ N) may have sufficient plasticity in their response to phenological cues, such that individuals may be capable of altering behavior based on the conditions they experience (Saino et al., 2007). This plasticity may enable short‐distance migratory species to shift the timing of their migration in response to the interannual variation in local conditions, as reflected by migration advancements being observed at a higher rate among short‐distance migrants (Buskirk et al., 2012; Hurlbert & Liang, 2012; Zaifman et al., 2017). For short‐distance migrants, any phenological shifts taking place in their wintering grounds may be consistent with those in their summer breeding site, based strictly on the proximity of the sites (Butler, 2003). Contrastingly, the distance between wintering and breeding sites of long‐distance migrants is significant enough that conditions are more likely to have little correlation. Thus, long‐distance migrants may inherently be less capable of adjusting the timing of their migration, relying solely on endogenous rhythms or the factors that entrain them (photoperiod; Marra et al., 2005). This differing capacity of response among short‐distance and long‐distance migrants will become more consequential with continued climate change, as any shifts in phenology due to changes in precipitation, temperature, and other climatic factors may only elicit changes in the timing of migration from a subset of species.

While species capable of shifting the timing of their migration may be at an advantage in terms of reproduction and survival (Miller‐Rushing et al., 2010), successful reproduction depends on a series of conditions. First, ecological cues must be indicative of the conditions the migrants are attempting to respond to, such as peak food availability (Moe et al., 2009). If a mismatch arises between cues and optimal breeding conditions, leading migrants to arrive either before or after peak food availability, breeding females may not have the energy needed to successfully fledge young (La Sorte et al., 2018). Reduced food availability and therefore energy intake could lead to decreased clutch sizes, smaller offspring and lower nest success rates (Jonzén et al., 2007). A second condition necessary for phenotypic plasticity of migratory species to be beneficial is for there to be relative consistency in these shifts, both temporally and spatially. If climate‐induced changes are hyper‐localized, or fluctuate greatly, it may be difficult for migrants to respond to this variability.

For grassland birds that rely on habitat managed or modified by humans, climate‐related phenological shifts may have implications beyond those directly related to food availability. Changes in precipitation and temperature are expected to impact agricultural growing seasons in terms of length, timing, and productivity (Linderholm, 2006; Olesen & Bindi, 2002) and will likely require changes to land management practices. The quality of an agricultural product is based on its nutritional value which peaks and then declines over the course of the growing cycle. With advancement of the start of the growing season, as well as intensified rate of growth (Olesen & Bindi, 2002), peak nutritional value will occur at a different time than historically typical. Whether in the agriculture or forestry products industry, harvesters will likely respond accordingly (Alig et al., 2002) and adjust the timing of harvest to allow for the most harvests and highest quality product.

Savannah Sparrows (*Passerculus sandwichensis)* and Bobolinks (*Dolichonyx oryzivorus)* are ground‐nesting grassland songbirds that use agricultural lands for breeding habitat throughout much of their breeding distribution, particularly in northeastern North America. Savannah Sparrows are short‐distance migrants, traveling approximately 1,200 km each fall to the wintering grounds, as the eastern population likely centers around North and South Carolina (Woodworth et al., 2016); contrastingly, Bobolinks are long‐distance migrants, traveling on average 9000–9500 km to Bolivia and Argentina from their summer breeding grounds (Renfrew et al., 2013). By using hay and pasture lands, reproductive success and therefore population dynamics of both species are broadly influenced by the mowing and grazing schedule of farmers (Perlut et al., 2008). The overlap in timing of breeding and peak harvest for hay crops results in the breeding habitat of many Bobolinks and Savannah Sparrows being destroyed, sometimes repeatedly, over the course of a breeding season (Perlut et al., 2006). Birds that nest on fields harvested before June 11 have a 99% chance of nest failure (Perlut et al., 2008). Importantly, in the Champlain Valley of Vermont and New York, hay farmers reported that their seasonal harvests advanced in timing and increased in frequency over the past 30 years (Troy et al., 2005). Although the cause of this shift is not clear, it can potentially be linked to a more favorable shift in temperature and precipitation and therefore growing season (Fei et al., 2017) making hay ready to harvest earlier than historically observed. If changes in harvesting times and frequency can be attributed to shifts in growing seasons, these changes may become more dramatic with climate change (Munson & Long, 2016).

Because of the relative proximity of the Savannah Sparrow wintering and breeding grounds, climatic‐driven changes are likely to be similar among their seasonal habitats, although this is unlikely the case for Bobolinks wintering in the southern hemisphere. Due to the differential effects of climate change on each region, shifts that occur in the wintering grounds and stopover sites of Bobolinks in South America may be drastically different from those occurring on the breeding grounds (Guilbert et al., 2014; Marengo et al., 2013). Using our long‐term demographic dataset (2002–2019), we explored how meteorological conditions on the wintering grounds, breeding grounds, and stopover sites explained variation in both nest initiation and nest survival in Bobolinks and Savannah Sparrows breeding in agricultural fields in Vermont. Furthermore, we explored if climatic variation in these regions also explained variation in the timing of hay harvest. We predicted that winter conditions experienced by the short‐distance migrant, the Savannah Sparrow, but not the long‐distance migrant, the Bobolink, would explain the timing and success of nesting, however that this timing would be misaligned with changes in agricultural practices by hay farmers.

## 2 METHODS

### 2.1 Nest and haying data

In 2002–2019, we monitored Savannah Sparrows and Bobolinks from mid‐May to early‐August in agricultural grasslands in the Champlain Valley of Vermont and New York, USA. We collected data on seven primary hayfields that ranged in size from 16.3 ha to 19 ha. Our study fields were in five treatment types: traditional early‐hayed fields were cut between 16 May and 11 June and generally again 35 to 52 days later, grassland bird incentive fields were cut before May 29 and had a 65‐day window between the first and second cuts, middle‐hayed fields were hayed after 21 June, late‐hayed fields were cut after 1 August, typically after most birds have ended their reproductive season, and rotationally grazed pastures (see Perlut et al., 2006, 2011 for additional details on field management).

Our field methods followed Perlut et al., 2006. In mid‐May, we spent one to two days with 30–35 12‐m mist nets capturing breeding adults on each study field. We banded each adult with three plastic color bands and one metal US Geological Survey band. We then visited each field every one to two days and searched for nests (1–3 people per field per day), typically using adult behavioral cues to find them. If either the male or female for an associated nest was unbanded, we immediately captured that individual near the nest and banded them. For our study population of both Bobolinks and Savannah Sparrows, the length of the average incubation period is ten days and one egg is laid per day, such that the initiation date = hatch date—10—# eggs laid. We were interested in each female's first nest attempt; therefore, the dataset excluded 1) all nests confirmed to be an individual's second or later attempt, and 2) all nests initiated after June 10, as these are second nest attempts (see Perlut et al., 2006). We recorded the average first mowing dates of all study fields for 2002–19.

### 2.2 Historical climate

To determine the specific wintering grounds for this study population of Bobolinks, we used data from light‐level geolocators which revealed that Bolivia and Argentina are the population's wintering grounds and Venezuela is a stopover site (Renfrew et al., 2013). More specifically, we used Santa Ana, Bolivia, Resistencia, Argentina and Barquisimieto, Venezuela as the approximate wintering and stopover locations of the Bobolinks based on geolocator data, as well as the fairly limited availability of historical climate data. Information regarding the specific wintering location of Savannah Sparrows was based largely on a study following a population that breeds on Kent Island, NB (515 km W of our study sites). The Kent Island population showed a significant distribution difference of approximately 275 km between males and females, with a midpoint at Wilmington, North Carolina (Woodworth et al., 2016). Because this research suggested that female Savannah Sparrows winter 275 km south of male Savannah Sparrows, the locations of Georgetown, South Carolina and Goldsboro, North Carolina, which are approximately 137 km SSW and 137 north of Wilmington, respectfully, were used as proxies for male and female wintering grounds weather and climate data. We did not consider wintering locations beyond those thought to be consistent with our study population.

We gathered historical weather information, including average daily maximum temperature and average daily precipitation for all seven locations using the Weather Underground public weather resource (https://www.wunderground.com/history). We used average maximum temperature as it correlates the timing of migration with arrival date, departure date, and duration of stays in breeding or wintering grounds (Zaifman et al., 2017). The temperature and precipitation data used for each site were only for the respective time span in which the species is known to be present. For Bobolinks, we used weather data for the months of October through November (Venezuela stopover), November through December (Bolivia stopover), January through March (Argentina), and May through August (Vermont). For Savannah Sparrows, we used weather data for the months of November through March for all three wintering locations and for the months of May through September for Vermont. We also used temperature and precipitation data for the month of April (following Woodworth et al., 2016) for each of the three Savannah Sparrow wintering locations as these conditions may be important in the timing of departure for the breeding grounds.

Given the transoceanic migration of Bobolinks, it is more complicated to understand the weather conditions they face during spring migration, and as a result, the factors that may be determining the timing of their departure. In addition to temperature and precipitation data at four Bobolink wintering and stopover regions, we also used historical data on larger climatic phenomena, including the North Atlantic Oscillation (NAO) and El Niño Southern Oscillation (ENSO); these data represent the potential large‐scale meteorological impacts on long‐distance migrants (Marra et al., 2005).

The NAO index is a teleconnection pattern caused by the difference in surface sea‐level pressure between the Subtropical (Azores) High and the Subpolar Low, which tend to fluctuate seasonally. In the positive phase of NAO, low pressure across the Icelandic region and Arctic interact with high pressure across the subtropical Atlantic and collectively produce stronger‐than‐average westerlies across mid‐latitudes (Visbeck et al., 2001). Positive phases of NAO are associated with colder and drier than average conditions over the northwestern Atlantic and Mediterranean regions and warmer and wetter than average conditions in northern Europe and the eastern United States. Contrastingly, NAO’s negative phase, which occurs when the subpolar low and subtropical high pressure are weaker than average, the Atlantic jet stream moves more west‐to‐east, resulting in increased storm frequency, above‐average precipitation and warmer temperatures in southern Europe and the eastern United States (Dahlman, 2009). These phases are generally used to explain severe weather conditions and were considered in this study to account for factors that may have been particularly influential if encountered during migration by both species. Historical data for the NAO index were collected from the National Oceanic and Atmospheric Administration (https://www.ncdc.noaa.gov/teleconnections/nao/).

### 2.3 Future climate

We used the KNMI Climate Explorer tool to extract predicted future temperature and precipitation for our study regions (https://climexp.knmi.nl/start.cgi). This tool uses the Coupled Model Intercomparison Project Phase 5 (CMIP5) which is the dataset used by the Intergovernmental Panel on Climate Change's Work Group One Fifth Assessment Report (IPCC WG1 AR5). Due to the current level of development of this projection tool, the spatial resolution is coarser than the geolocator data. Moreover, grids covering 2.5º x 2.5º in longitude and latitude were used to project future precipitation and temperature in four different greenhouse gas concentration scenarios as represented by four representative concentration pathways (RCP’s), 2.6, 4.5, 6, and 8.5. The number associated with each RCP corresponds to the radiative forcing at which the atmosphere will stabilize under the respective greenhouse gas concentration. These four pathways are inclusive of a wide range of scenarios regarding atmospheric greenhouse gas levels, including that which would result from a continuation of the current rate of emissions to levels that would result from aggressive carbon mitigation efforts. Because of the complexity and variability of NAO and ENSO, as well as the structure typical of climate prediction systems, there is a great deal of uncertainty regarding the future of NAO and ENSO in the context of a changing climate (Guilyardi, 2015; Siegert et al., 2016). The projections used in our models to predict nest initiation and nest survival assume that both NAO and ENSO will follow the same trends as has been recorded since the initiation of our study in 2001. This assumes seasonal and annual variation, for NAO and ENSO, respectively, which is considered in our data as interactions with other climatic factors.

### 2.4 Statistical analysis

We used an information‐theoretic approach (Burnham & Anderson, 2002) to compare and rank alternative models to determine which climatic factors, including additive and two‐way interactive models best explained variation in daily nest survival and nest initiation date (see Table 1 for list of all factors included in the model sets). We ran daily nest survival models in program MARK (Dinsmore & Dinsmore, 2007; White & Burnham, 1999). Program MARK uses a numerical maximum likelihood technique to provide parameter estimates from repeated nest encounters (visits). For understanding variation in nest initiation date, we ran with two‐way ANOVA tests in PROC MIXED (SAS Institute, Cary, North Carolina, USA). In both analyses, we ranked models using Akaike's information criterion adjusted for small sample sizes (AIC_c_). We considered strongly supported models to have a ΔAIC_c_ of <2 and moderately supported models to have a ΔAIC_c_ between 2 and 4 (Anderson 2008). We interpreted biological significance within the top‐ranked models (ΔAIC_c_ ~ 2) by examining beta values and their associated 95% confidence intervals. We considered factors whose 95% confidence interval did not include zero as biologically significant. Finally, we ran two‐way ANOVA tests in PROC MIXED to see if the factors in the top‐ranked nest initiation and daily nest survival models for both species explained variation in haying dates.

**TABLE 1:** Factors used in Program MARK models to explain variation in daily nest survival and nest initiation date for Bobolinks and Savannah Sparrows nesting in the Champlain Valley of Vermont, USA

Location	Model factor	Species
Breeding grounds	Burlington average precipitation	both
	Burlington average temperature	
	NAO August	
Spring migration	Georgetown (female) average April precipitation	Savannah Sparrow
	Georgetown (female) average April temperature	
	Goldsboro (male) average April precipitation	
	Goldsboro (male) average April temperature	
	Wilmington average April precipitation	
	Wilmington average April temperature	
	Venezuela average temperature	Bobolink
	Venezuela average precipitation	
Wintering	ENSO December–February	both
	ENSO January–March	
	Georgetown (female) average precipitation	Savannah Sparrow
	Georgetown (female) average temperature	
	Goldsboro (male) average precipitation	
	Goldsboro (male) average temperature	
	NAO November–March	both
	Wilmington average precipitation	Savannah Sparrow
	Wilmington average temperature	
	Argentina average temperature	Bobolink
	Argentina average precipitation	
	Bolivia average temperature	
	Bolivia average precipitation	
Winter–spring migration	ENSO February–April	both

## 3 RESULTS

**FIGURE 1:**
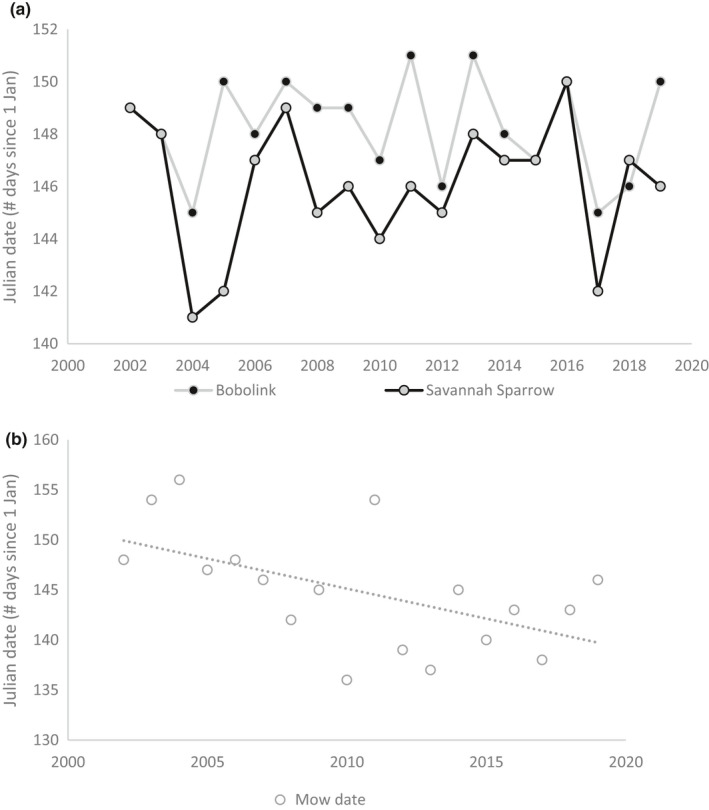
Nest initiation date (Julian date) of Savannah Sparrows and Bobolinks (a) and first hay harvest dates (b) from 2002 to 2019 in the Champlain Valley of Vermont

We found 634 and 598 Savannah Sparrow and Bobolink nests, respectively, in 2002–2019. The mean (±*SD*) first clutch initiation dates were May 28 (±1.9) and May 26 (±2.4) for Bobolinks and Savannah Sparrows, respectively (Figure 1). For Savannah Sparrows, the interaction between precipitation during the breeding season (Burlington, VT) and precipitation in the mean wintering region (Wilmington, NC) best explained variation in nest initiation, as this model explained nearly all of the variation in the dataset (*weight (w)* = 0.97; Table 2). For Bobolinks, the interaction between precipitation during the breeding season (Burlington, VT, USA) and average temperature at their primary South American fall migration stopover site (Barquisimieto, VZ) best explained variation in nest initiation (*w* = 0.94, Figure 1, Table 2).

**TABLE 2:** All candidate nest initiation models with ∆AIC_c_ <2.00 for Bobolink and Savannah Sparrow nest initiation dates with AIC_c_ weights (w), Champlain Valley, USA, 2002–2019 (AIC, Akaike's information criterion)

Savannah Sparrow			
Model	∆AIC_c_	# parameters	AICc weight (*w*_i_)
Wilmington precipitation * Burlington precipitation	0	4	0.97
Bobolink			
Model	∆AIC_c_	# parameters	AIC_c_ weight (*w*_i_)
Burlington precipitation*Venezuela average temperature	0	4	0.94

For Savannah Sparrows, variation in daily nest survival was explained by five models with a ΔAIC_c_ of <2.0 (Table 3). The top ranking model included the interaction between April precipitation in the mean wintering region (Wilmington, NC) and ENSO values for February through April (*w* = 0.14). The beta values and confidence intervals indicate that the NAO in April and the interaction factor between NAO April and precipitation on the breeding grounds was biologically significant (Table 4). The second‐ranked model included the interaction between average temperature for the mean female wintering region (Georgetown, SC) and the NAO index for November through March (*w* = 0.11). For Bobolinks, variation in nest survival was explained by three models with a ΔAIC_c_ of <2.0 (Table 3). The top‐ranked model included the interaction between the NAO index for April and precipitation during the breeding season (Burlington, VT; *w* = 0.17). Both factors, and their interaction, were biologically significant (Table 4). The second‐ranked model included the interaction between the NAO index in August and the average temperature at the northern wintering location (Santa Anna, BV; *w* = 0.12; Table 3).

**TABLE 3:** All candidate models with ∆AIC_c_ <2.00 for Bobolink and Savannah Sparrow daily nest survival with AIC_c_ weights (*w*_i_), Champlain Valley, USA, 2002–2019 (AIC, Akaike's information criterion)

Model	∆AIC_c_	# of parameters	AIC_c_ weight (*w*_i_)
Savannah Sparrow			
Will Apr precip * ENSO FMA	0	4	0.14
Female April avg temperature * NAO November to March	0.6	4	0.11
Will precip +Burl avg temperature	1.1	3	0.08
Will April avg temperature * ENSO January to March	1.24	4	0.08
Will precip +NAO August	1.98	3	0.05
Bobolink			
NAO April * Burlington precip	0	4	0.17
NAO Aug * Bolivia avg temperature	0.7	4	0.12
Argentina avg temperature * Burlington avg temperature	0.74	4	0.12

**TABLE 4:** Parameter estimates for daily nest survival models for Bobolinks and Savannah Sparrows breeding in Vermont, USA. Confidence intervals (LCI =lower, UCI =upper) that do not cross zero are considered biologically significant

Species	Model	Parameter	Beta	SE	LCI	UCI
Bobolink	NAO April * Vermont precipitation	Intercept	2.67	0.38	1.92	3.42
		Burlington precipitation	6.58	2.61	1.46	11.71
		NAO April	1.18	0.45	0.30	2.05
		Interaction	−10.51	3.53	−17.42	−3.60
Savannah Sparrow	Wilmington April precipitation * ENSO February to April	Intercept	3.37	0.13	3.12	3.62
		Wilmington April precipitation	−1.19	0.97	−3.10	0.71
		ENSO February to April	1.07	0.24	0.61	1.53
		Interaction	−9.35	2.36	−13.98	−4.72

First hay harvest dates across our study region advanced significantly over the 18‐year period from ~30 May to ~20 May (*F*_1,16_ = 7.52; *p* = 0.0145; Figure 3). This change did not correspond with any of the models that explained either nest initiation date or daily nest survival for either Savannah Sparrows or Bobolinks (Table 5).

**TABLE 5:** The models that best explained variation in nest initiation and daily nest survival in Bobolinks and Savannah Sparrows did not explain first haying dates for the Champlain Valley of Vermont

Model	F	*df*	p
Wilmington April precipitation * ENSO February–April (Savannah Sparrow)	3.47	14	0.08
Burlington precipitation * NAO April (Bobolink)	0.18	14	0.67
Burlington precipitation * Venezuela average temperature (Bobolink)	1.22	13	0.29
Wilmington precipitation * Burlington precipitation (Savannah Sparrow)	0.71	14	0.42

**FIGURE 2:**
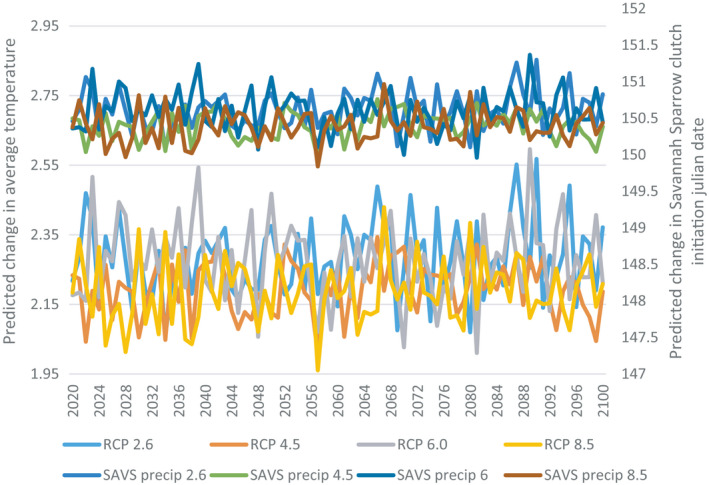
Future projections of Savannah Sparrow (SAVS) nest initiation date in Vermont and the interaction between Wilmington and Burlington precipitation (SAVS precip). Future precipitation and temperature in four greenhouse gas concentration scenarios are represented by four representative concentration pathways (RCP’s), 2.6, 4.5, 6, and 8.5. The number associated with each RCP corresponds to the radiative forcing at which the atmosphere will stabilize under the respective greenhouse gas concentration

**FIGURE 3:**
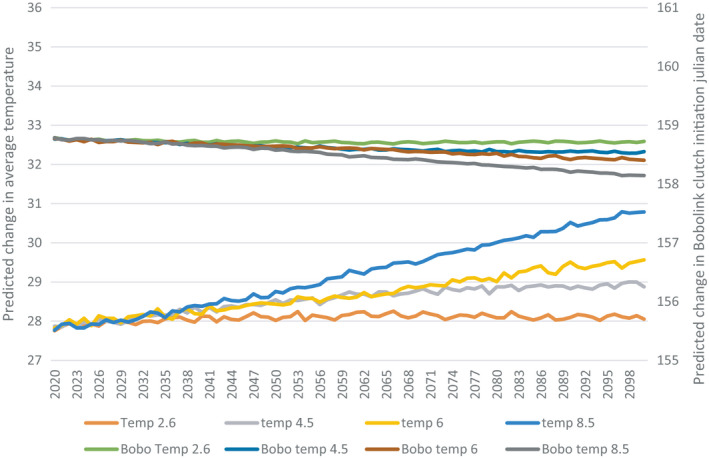
Future projections of Bobolink (BOBO) nest initiation date and the interaction between average temperature in Venezuela and precipitation in Vermont, USA. Future precipitation and temperature in four greenhouse gas concentration scenarios are represented by four representative concentration pathways (RCP’s), 2.6, 4.5, 6, and 8.5. The number associated with each RCP corresponds to the radiative forcing at which the atmosphere will stabilize under the respective greenhouse gas concentration

Under all greenhouse gas concentration scenarios, weather variables from the wintering grounds that best explained variation in nest initiation date are predicted to change notably by the year 2,100 (Figures 2, 3). However, based on the results of our models, neither species is predicted to advance or delay their nest initiation date.

## 4 DISCUSSION

Although the weather and climactic factors from the migratory and wintering grounds that explained variation in the nest initiation date of Savannah Sparrows and Bobolinks are projected to change in the context of a changing climate, manifesting as increased temperatures and more variable precipitation among all four representative concentration pathways (RCP’s), our results suggest that there will be no significant change in nest initiation date (Figures 2 and 3). Moreover, Savannah Sparrows and Bobolinks are unlikely to respond to phenological shifts by adjusting timing of nest initiation. Other studies have attributed interannual variation in nest initiation to ENSO and NAO (Miller‐Rushing et al., 2008). While there is annual variation in nest initiation date among both Savannah Sparrows and Bobolinks, the long‐term trend is flat for both species (Figures 3 and 4). However, there has been a notable advancement in the timing of haying among dairy farmers in the Champlain Valley of Vermont. Further, the factors which were determined to be most influential on the nest initiation and nest survival of both Savannah Sparrows and Bobolinks did not influence the timing of haying (Figure 3). Collectively, this differential response between migratory birds and farmers to phenological shifts driven by climate change suggests that the previously observed mismatch between haying and nesting are likely to persist, and potentially worsen, in the future. Farmers who now typically cut later in the season will likely advance their first cut into the core breeding season and farmers who cut early will cut a second time, also in the core breeding season. The existing comprehensive haying guidelines (Perlut et al., 2011), while currently serving to correct the mismatch between haying and nesting, may no longer be effective, resulting in a further decline in nest success. If farmers continue to advance the timing of their first haying consistent with what was observed over the 18 years of this study, birds that nest on what is currently considered a late‐hayed field may no longer have a reproductive advantage over birds that lay on early‐ and mid‐hayed fields. If the observed advancement of haying is triggered by the advancement of the growing season, hay crops will reach their nutritional peak earlier, and farmers may need more financial incentive than that which is currently offered to adjust the timing of their second harvest.

Shifts in migration timing have been observed among many species of birds, including long‐ and short‐distance migrants, many of which have been attributed to climate change and related phenological changes (Hüppop & Hüppop, 2003; Zaifman et al., 2017). In some cases, short‐distance migrants have adjusted the timing of their migration as compared to long‐distance migrants (Butler, 2003, Both et al., 2006). This trend has been explained by the commonly held theory that short‐distance migrants are more plastic in their migration than long‐distance migrants who are thought to rely strictly on innate rhythms rather than environmental factors (Marra et al., 2005, Saino et al., 2007, but see Jonzén et al., 2006). There is, though, evidence of long‐distance migrants adjusting the timing of nest initiation relative to breeding grounds temperature (Hoover & Schelsky, 2020) and relative to the snow conditions on the spring migratory path (Oliver et al., 2020). While our models for Bobolinks, a long‐distance migrant, may be consistent with projections for other long‐distance migrants (Miller‐Rushing et al., 2008), our model results for Savannah Sparrows, a short‐distance migrant, are surprising. Considering the relative proximity between a Savannah Sparrow's wintering habitat and summer breeding grounds, Savannah Sparrows should be more flexible in their ability to adjust the timing of their migration to respond to climatic‐induced phenological changes (Pulido & Widmer, 2005). The results of our models suggest that the most recently accepted theory regarding differential response, or lack thereof, between long‐distance and short‐distance migrants does not apply to the case of Bobolinks and Savannah Sparrows.

The possible explanations for the lack of directional change predicted in timing of nest initiation among both Savannah Sparrows and Bobolinks may be unrelated to climate change. While variation in nest initiation date was explained by climatic factors, nonclimatic factors not considered in our models, such as disruption during migration and competition for mates or territories, may be even more influential on nest initiation. Moreover, factors other than ecology and life‐history traits may play a role in determining migratory behaviors and may help to explain the lack of change predicted by our models, as well as decreased migration distance or duration, observed among other species (Bókony et al., 2007). For example, habitat type, habitat urbanization and anthropogenic food supplementation, may play a significant role in these species nest initiation date (Bókony et al., 2007). Another potential explanation of projected consistency in nest initiation date is response to phenological change manifesting as a spatial shift rather than a temporal one. Many migratory bird species have been observed to shift their wintering grounds northward in response to increased temperatures and milder winters (Hitch & Leberg, 2007). If conditions allow more northward wintering and therefore shorter spring migrations, migrants would be able to reduce a taxing and dangerous component of their annual cycle (Visser et al., 2001). For Savannah Sparrows, shorter distances between wintering and breeding grounds could allow for more consistency in conditions between habitats and potentially reduced urgency for spring migration.

Alternatively, the projections of consistent timing of nest initiation of both Savannah Sparrows and Bobolinks may be indicative of more extreme interspecific response to climate change and related phenological shifts. While the widely accepted theory focuses on the differential migratory response is based on the categorization of short‐ and long‐distance migrants, phenotypic plasticity may vary on a species‐by‐species basis. The stronger the endogenous control of migration, the less flexible and therefore more consistent migration will be (Bókony et al., 2007).

While climate change and the associated phenological shifts pose a threat for many migratory species, the interaction between grassland birds and agricultural activity introduces an additional level of threat for Bobolinks and Savannah Sparrows. They are susceptible not only to biological shifts, such as changes in food availability (but see Zalik & Strong, 2008) due to shifts in insect diapause and fruit availability, as well as potential changes in landscape and loss of wet grasslands due to decreased and more variable precipitation (Renfrew et al., 2019) but also to anthropogenic shifts, such as the of timing of haying. For the Bobolink, whose population is far less stable than that of the Savannah Sparrow, this multifaceted threat is particularly problematic. Bobolink populations, nationally, are predicted to decline by 50% in the next 48 years due to the pressures facing them in all of their habitats (Renfrew et al., 2019). To conserve this fragile species, a full life cycle conservation approach must be used to address threats in all of their habitats.

Without aggressive intervention with the current rate of greenhouse gas emissions, climate change is forecasted to persist into the future, resulting in increased temperatures and more variable and extreme precipitation (IPCC, 2007). Given the consistency of Bobolink and Savannah Sparrow nest initiation dates, we can reasonably predict that their fitness will eventually be impacted by climate‐induced interactions between nest initiation dates and the timing of hay harvest. To most effectively protect these species, particularly the already declining Bobolink, we must alleviate other stressors that are more easily controllable, such as haying date. By continuing to monitor and analyze haying activity and any directional trends in timing, we can effectively inform haying incentives and policy to adequately consider the reproductive needs and cycles of grassland birds in the context of a changing climate.

## CONFLICT OF INTERESTS

There are no conflicting interests to report.

## AUTHOR CONTRIBUTIONS

**Noah Perlut:** conceptualization, formal analysis, funding acquisition, investigation, methodology, project administration, software, supervision, validation, writing—review and editing (lead), visualization (supporting). **Maeve McGowan:** conceptualization, data curation, funding acquisition, investigation, (supporting), resources, visualization, writing—original draft preparation (lead). **Allan Strong:** writing—review and editing (equal).

## Data Availability

Climate data and reproductive data input files: Dryad (https://doi.org/10.5061/dryad.2280gb5rm).
